# How chrysanthemum (*Chrysanthemum × grandiflorum*) ‘Palisade White’ deals with long-term salt stress

**DOI:** 10.1093/aobpla/plac015

**Published:** 2022-04-02

**Authors:** Hanna Bandurska, Włodzimierz Breś, Agnieszka Tomczyk, Małgorzata Zielezińska, Klaudia Borowiak

**Affiliations:** 1 Department of Plant Physiology, Poznan University of Life Sciences, Wołyńska 35, 60-637 Poznań, Poland; 2 Department of Plant Nutrition, Poznan University of Life Sciences, Zgorzelecka 4, 60-198 Poznań, Poland; 3 Department of Ecology and Environmental Protection, Poznan University of Life Sciences, Piątkowska 94C, 60-649 Poznan, Poland

**Keywords:** Chloroplast pigments, growth, membrane injury, photosynthesis, proline, potassium

## Abstract

Salinity is a serious problem in the cultivation of ornamental plants. Chrysanthemum (*Chrysanthemum × grandiflorum*) ‘Palisade White’ was evaluated in order to examine its responses to long-term salt stress. Plants were grown in substrate supplemented with NaCl doses (g dm^−3^ of substrate) 0, 0.44, 0.96, 1.47, 1.98, 2.48 and 2.99. The initial electrical conductivity (EC) of the substrates was 0.3, 0.9, 1.4, 1.9, 2.6, 3.1 and 3.9 dS m^−1^, respectively. Plant growth, relative water content (RWC), Na, Cl, K, N and P concentrations, membrane injury (MI), chlorophyll and proline levels, as well as gas exchange parameters in leaves of chrysanthemum were determined. A dose-dependent significant reduction of growth and minor decrease of leaf RWC were observed. Foliar Na and Cl concentrations increased with the highest NaCl dose up to 6-fold. However, the concentration of K increased by about 14 %, N by about 5 % but P decreased by about 23 %. Membrane injury was rather low (11 %) even at the highest NaCl dose. Statistically significant decreases of stomatal conductance (20 %), transpiration rate (32 %) and photosynthesis (25 %) were already observed at the lowest NaCl dose and about 40 % decrease of all these parameters with the highest dose. A significant reduction in the intercellular CO_2_ concentration occurred at the lower NaCl doses and no changes with the highest dose. These results show that in plants grown with the highest NaCl dose, non-stomatal limitation of photosynthesis may occur. According to Maas and Hoffman tolerance assessment (1977) chrysanthemum ‘Palisade White’ may be considered as moderately sensitive to salt stress in terms of growth inhibition. However, it is able to cope with long-term salt stress without any signs of damage, such as chlorophyll depletion, leaf browning or necrotic spots probably due to maintenance of K homeostasis and proline accumulation, which alleviate the toxic effect of chloride.

## Teaser

Plant species and cultivars vary in their sensitivity to salinity caused by sodium chloride. The aim of our study was the evaluation of the physiological and biochemical responses of *Chrysanthemum*× *grandiflorum*‘Palisade White’ to the long-term salt stress. Salinity stress caused photosynthesis limitation and plant growth inhibition. Undisturbed potassium uptake and synthesis of amino acid proline probably help plants to deal with salt stress by protecting against its harmful effect. The research has shown that the examined chrysanthemum is moderately resistant to salt stress and is suitable for cultivation in slightly saline substrates.

## Introduction

Soil and substrate salinity is a serious problem in the horticulture industry, especially in the cultivation of cut flowers, potted flowering plants, bedding plants and herbaceous perennials (Cassaniti *et al.* 2012). It is caused by large amounts of salts (e.g. NaCl) which are often present in compost made from biowaste in the production of potting substrates for horticulture (Cai and Gao 2011). Salt accumulation in soil and substrate during the cultivation of floricultural crops is also an effect of reuse of wastewater for irrigation (Cassaniti *et al.* 2013; Elhindi 2013). According to the USDA Salinity Laboratory, saline soil is defined as soil which has an electrical conductivity (EC) of the saturation extract at the level of 4 dS m^−1^ (equivalent to approximately 40 mM NaCl) or more (Munns and Tester 2008). Four classes of soil salinity are distinguished. Electrical conductivity at the level of 1.5 to 2 dS m^−1^ indicates slightly saline soil, moderately saline soil is characterized by EC from 2 to 6 dS m^−1^, EC from 6 to 15 dS m^−1^ means highly saline and extremely saline corresponds to EC exceeding 16 dS m^−1^ (Yadav *et al.* 2011).

Chrysanthemum is a well-known floricultural crop cultivated for cut flowers, pot flowers and garden plants. In the literature there is no clear evaluation of chrysanthemum sensitivity to salt stress. Differences in salt resistance are observed among chrysanthemums species as well as varieties. Chen *et al.* (2003) examined the resistance to salinity in three chrysanthemum genotypes and found that the most sensitive was *Chrysanthemum indicum* ‘Hangzou’ compared to *C. indicum* ‘Nanjong’, which was more tolerant than *Chrysanthemum chanetii.* Out of two chrysanthemum wild species *Chrysanthemum okiense* was more salt-tolerant than *Chrysanthemum ornatum* (Guan *et al.* 2012). Rahi and Singh (2011) stated that *Chrysanthemum morifolium*, which is a plant of halophytic origin, can be grown successfully in a moderately saline environment. However, *C. morifolium* ‘Amiko red’ proved to be negatively affected by salt stress even at low (10 mM) NaCl concentrations (Paraskevapoulu *et al.* 2020). *Chrysanthemum morifolium* ‘Balady’ irrigated with saline water up to 4 dS m^−1^ showed good salinity resistance. What is more, plants grown in zeolitic tuff coped better with higher salinity than those grown in soil (Amarin *et al.* 2021). Rai *et al.* (2017) observed significant differences in salinity tolerance between 22 varieties of *C. morifolium* and grouped them into tolerant, moderately tolerant, susceptible and highly susceptible, with the largest group (16 varieties) in the category of moderately tolerant.

Increased salt concentration in soil or substrate causes osmotic and ionic stress. Osmotic stress is a result of the decrease in soil water potential, which reduces the ability of roots to take up water, leading to the restriction of cell elongation, stomatal closure and photosynthesis inhibition (Munns and Tester 2008; Roy *et al.* 2014; Parihar *et al.* 2015). Ionic stress, on the other hand, is caused by excessive amounts of salts which enter the plant and have a detrimental effect on DNA, enzyme activity, cell membrane stability, the content of chloroplast pigments and photosynthetic rate (Liang *et al.* 2018; Isayenkov and Maathuis 2019). Salt stress may also cause disturbance of the uptake of mineral ions, leading to nutrient starvation, and physiological activity of plants (Tavakkoli *et al.* 2011). Tip and marginal leaf burn caused by accumulation of toxic ions affects plants’ decorative value (García-Caparrós and Lao 2018). The range of deleterious effects of salt stress on plant metabolism and physiological processes depends on the salinity levels and the duration of stress. Plants have developed different responses to soil salinity which overcome the adverse effects of osmotic and ionic stresses (Parihar *et al.* 2015; van Zelm *et al.* 2020). One such response is the accumulation of free proline, which plays an important role in resistance to salinity (Munns and Tester 2008). In addition to the participation in osmotic adjustment, proline acts as an enzyme protectant, free radical scavenger, cell redox balancer and stabilizer of subcellular structure (Per *et al.* 2017; Meena *et al.* 2019). The beneficial effect of the application of exogenous proline on salinity resistance was revealed in many crops (Maukhtari *et al.* 2020 and reference therein). Certain recent research has shown that the ameliorative effect of proline on mitigating the detrimental effect of salt stress is probably related to the improvement of ionic homeostasis, maintaining a favourable leaf water content and chloroplast pigment concentration (Rady *et al.* 2016; de Freitas *et al.* 2019).

The aim of the present study was to evaluate the responses of chrysanthemum (*Chrysanthemum × grandiflorum* Ramat./Kitam.) to long-term salt stress, imposed by the addition of increased NaCl doses to the substrate. The applied NaCl doses were chosen based on the salinity level found in the substrate used for the cultivation of floricultural crops. The effects of salt stress on relative water content (RWC), Na, Cl, K, N, P, chlorophyll and proline concentrations in leaves, as well as on the membrane injury (MI) index, were examined. Plant performance under salt stress conditions was assessed by measuring the net photosynthetic rate (*P*_N_), intercellular CO_2_ concentration (*C*_i_), stomatal conductance (*g*_s_), transpiration rate (*E*) and growth parameters (leaf number and area, plant height, stem growth rate, fresh matter).

## Materials and Methods

### Plant cultivation and treatments

Two independent experiments were conducted during two vegetation seasons. In each season plants of the chrysanthemum ‘Palisade White’ were cultivated from July to the end of October under greenhouse conditions at the Experimental Station of Poznań University of Life Sciences (16°54ʹE, 52°23ʹN). Plants were grown under natural light conditions without additional lighting. Throughout the experiment the average day/night temperatures in the greenhouse were 24.5/14 °C and 23/13.5 °C in experiment 1 and 2, respectively. Average relative humidity was at the level of 75 % in experiment 1 and 79 % in experiment 2. Sphagnum peat (Hartman, pH 4.33) was used as a growing medium. Lime was used (CaCO_3_ and MgCO_3_) in order to obtain pH 6.5. After liming the concentration of Ca and Mg in the growing medium was 1245 mg dm^−3^ and 160 mg dm^−3^, respectively. The concentration of NPK was supplemented, providing the following levels (mg dm^−3^): N-NO_3_ 300, P 180, K 400 using KH_2_PO_4_, KNO_3_ and NH_4_NO_3_. The polymer Polichelat LS-7 was used for micronutrient supplementation to the fallowing levels (mg dm^−3^): Fe 75, Mn 23, Zn 30, Cu 20, B 1.0 and Mo 1.0. Additional supplementation with 50 mg N-NO_3_ mg dm^−3^ was applied at the beginning of the generative stage.

The experiments included six doses of sodium chloride (g dm^−3^ of substrate): 0.44, 0.96, 1.47, 1.98, 2.48 and 2.99 g dm^−3^, which corresponds to 7.5, 16.4, 25.2, 33.9, 42.4 and 51.2 mM of NaCl, respectively. The seventh combination was the control with no salt added. The initial EC of the substrates was: 0.9, 1.4, 1.9, 2.6, 3.1, 3.9 dS m^−1^ (saline treatment) and 0.3 dS m^−1^ (control). Electrical conductivity of growing medium was measured using an Orion Benchtop Conductivity Meter (Thermo Electron Corporation).

Rooted cuttings were transplanted into 4-dm^3^ pots filled with substrate in mid-July. Each treatment consisted of 20 pots with one chrysanthemum plant per pot. Water purified by reverse osmosis was used for irrigation. During the experiment moisture of the growing medium was kept at the level of about 60 % of capillary water capacity. At the end of October when half of the inflorescences were fully opened, plant material was collected to determine leaf number and area, stem growth rate, plant height, fresh matter, RWC, Na, K, Cl, N, P, chlorophyll and proline concentrations, as well as MI in leaves. Additionally, in experiment 2 gas exchange parameters were measured.

### Growth parameters

Leaf area was determined with the Leaf Area Meter CI-202 (CID BioSciences, USA). For this purpose, leaves from all plants located at the fourth node (counted from the top of the plant) were used. The rate of stem growth was estimated on the basis of the average daily increase of plant height, by dividing the final growth (height) by the time of cultivation. The results presented are means of 20 replications.

### Mineral concentration

Fully mature leaves were pre-dried at a temperature of 105 °C for 48 h and ground in a mixer (laboratory grinder 06-FW-135, Chemland, Poland). Plant material was mineralized with a mixture of H_2_SO_4_ and H_2_O_2_ (2:1), and the concentrations of Na and K were determined by flame emission spectrophotometry (FAAS, on a Carl Zeiss Jena apparatus). The concentration of N was determined by the micro-Kjeldahl procedure and P by spectrophotometric methods (UV-VIS, model 8001, Metertech Inc.). Plant material for Cl determination was mineralized at a temperature of 500 °C. The residue was dissolved in hot deionized water and, after sedimentation, the content of Cl was determined in clear solution by the nephelometric method (Kalra 1998). All analyses were carried out in three biological replicates. The results are expressed in grams per kg of dry matter (g kg^−1^ DM).

### Gas exchange parameters

Net photosynthetic rate (*P*_N_), stomatal conductance (*g*_s_), intercellular CO_2_ (*C*_i_) concentration and transpiration rate (*E*) were measured using a CI 340 handheld photosynthesis system (CID Bio-Science, Camas, WA, USA). The measurements were taken at the following constant conditions in the leaf chamber: CO_2_ inflow concentration 390 µmol mol^−1^, photosynthetic photon flux density 1000 µmol (photon) m^−2^ s^−1^, temperature 25 °C and relative humidity 40 ± 3 %. The measurements were carried out with nine independent replicates. Three uppermost fully expanded leaves from three plants were used for the measurements.

### Other physiological and biochemical parameters

Fully mature leaves taken from the fourth node (counted from the top of the plant) of five randomly chosen plants were used. Leaf water status and MI were estimated immediately after harvest. Plant material for the estimation of other parameters was frozen in liquid nitrogen and stored at −20 °C until analyses. The analyses were carried out using five independent biological replicates.

Leaf water status was estimated by measuring RWC, according to the standard method developed by Weatherly (1950) and was calculated using the following formula:


$$\begin{array}{l} RWC=\frac{fresh\;matter\;-\;dry\;matter}{fresh\;matter\;at\;full\;turgor-dry\;matter}\cdot 100 \end{array}$$

Membrane injury was estimated according to the method of Premachandra *et al.* (1990). Leaf pieces from plants of each combination were washed three times in 10 cm^3^ of deionized water and put into a 50-cm^3^ flask, submerged in 10 cm^3^ of deionized water and kept for 24 h at 10 °C. After warming to 25 °C and shaking, EC of the effusate was measured. Next, tissues were killed by autoclaving for 15 min, cooled down to 25 °C, and EC was measured once again. Membrane injury was evaluated according to the formula:


$$\begin{array}{l} MI=1-\;\frac{1-(T1/T2)}{1-\;(C1/C2)\;}\;\times \;100\% \end{array}$$

where *C*1 and *C*2 represent conductivity values of control samples before and after autoclaving, respectively; *T*1 and *T*2 represent conductivity values for NaCl-treated samples before and after autoclaving, respectively.

Total chlorophyll content was estimated according to the method of Hiscox and Israelstam (1979). Leaf samples (100 mg fresh matter) were cut into pieces and pigments were extracted at 65 °C using 5 cm^3^ of dimethyl sulfoxide. Optical density of extract was measured at 649 and 665 nm. The content of total chlorophyll was calculated following the modified Arnon equations (Wellburn 1994) and was expressed in milligrams per gram of dry matter (mg g^−1^ DM).

Proline was extracted and estimated as described by Bates *et al.* (1973) with some modification. Plant material (200 mg fresh matter) was homogenized with 4 cm^3^ of 5 % trichloroacetic acid (TCA v/v). The homogenate was centrifuged at 5000 *g* for 15 min. Supernatant was used for proline determination by measuring the quantity of the coloured reaction product of proline with ninhydric acid. The amount of proline was calculated from a standard curve and expressed in milligrams per gram of dry matter (mg g^−1^ DM).

### Statistical analysis

The experiments were carried out in a completely randomized design with 20 replications in each treatment. All collected data **[see****Supporting Information—Tables S1****–****S3****]** were submitted to statistical analyses. Statistical evaluation of the data was performed using STATISTICA 13.3 software (StatSoft, Inc., Tulsa, OK, USA). One-way analysis of variance (ANOVA) was used to determine whether salinity had a significant effect on estimated parameters. The *post hoc* Tukey test was performed to find significant differences between individual means of treatment groups in each experiment. The level of significance was set at *α* = 0.05. The differences were considered significant for *P* less than or equal to *α*. The relationships between stomatal conductance (*g*_s_), photosynthetic rate (*P*_N_) and transpiration rate (*E*) were also explored with Pearson correlation.

## Results

ANOVA results showed a significant effect of salt stress on all examined growth and physiological parameters (Table 1).

**Table 1.** On-way ANOVA results for the effect of salt dose on different parameters of *Chrysanthemum × grandiflorum* ‘Palisade White’. The level of significance is indicated by asterisks: *P < 0.05, **P < 0.01, ***P < 0.001; nd, not determined.

Source of variation	Df	Experiment 1	Experiment 2
*F*	*F*		
Number of leaves	6	132.17***	89.88***
Leaf area	6	62.49***	56.83***
Plant height	6	67.61***	97.69***
Stem growth rate	6	113.57***	86.85***
Fresh matter	6	211.40***	131.23***
RWC	6	18.30***	25.40***
Leaf N	6	8.3***	4.1*
Leaf P	6	9.67*	3.88*
Leaf Na	6	34.89***	48.91**
Leaf Cl	6	136.97**	129.21***
Leaf K	6	7.50***	28.90***
K:Na	6	46.42**	47.29**
Chlorophyll	6	4.87**	6.45***
MI	5	48.78***	164.48***
Proline	6	198.79***	138.77***
*g*_s_	6	nd	279.48***
*E*	6	nd	122.33***
*P*_N_	6	nd	175.12**
*C*_i_	6	nd	80.95***

### Growth parameters and leaf water status

**Figure 1.**
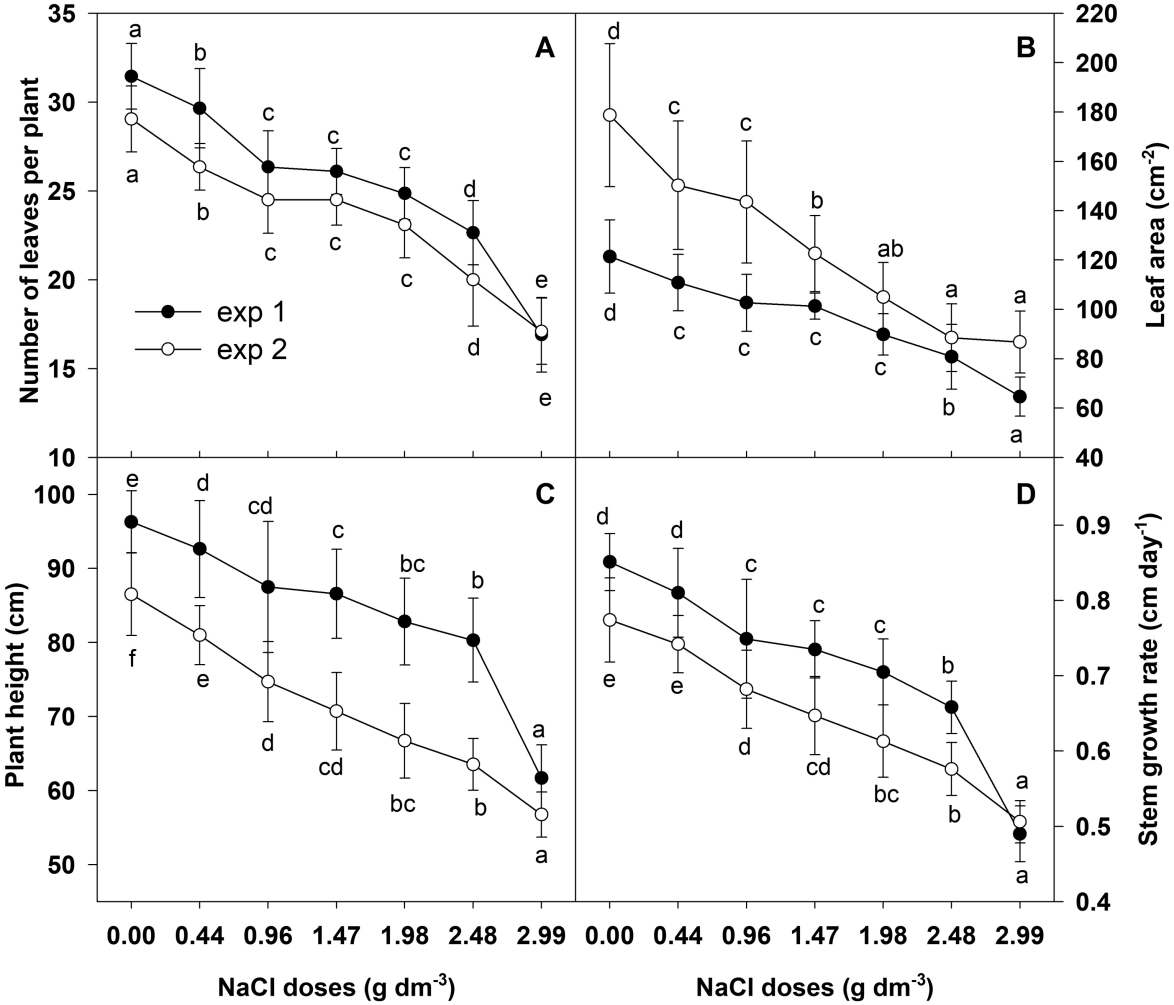
Effect of NaCl doses on growth traits of chrysanthemum. (A) number of leaves per plant, (B) leaf area, (C) plant height, (D) stem growth rate. Values are expressed as a mean ±standard deviation. Different letters across salinity treatments indicate significant differences between means (*n* = 20, Tukey test at *α* = 0.05).

**Figure 2.**
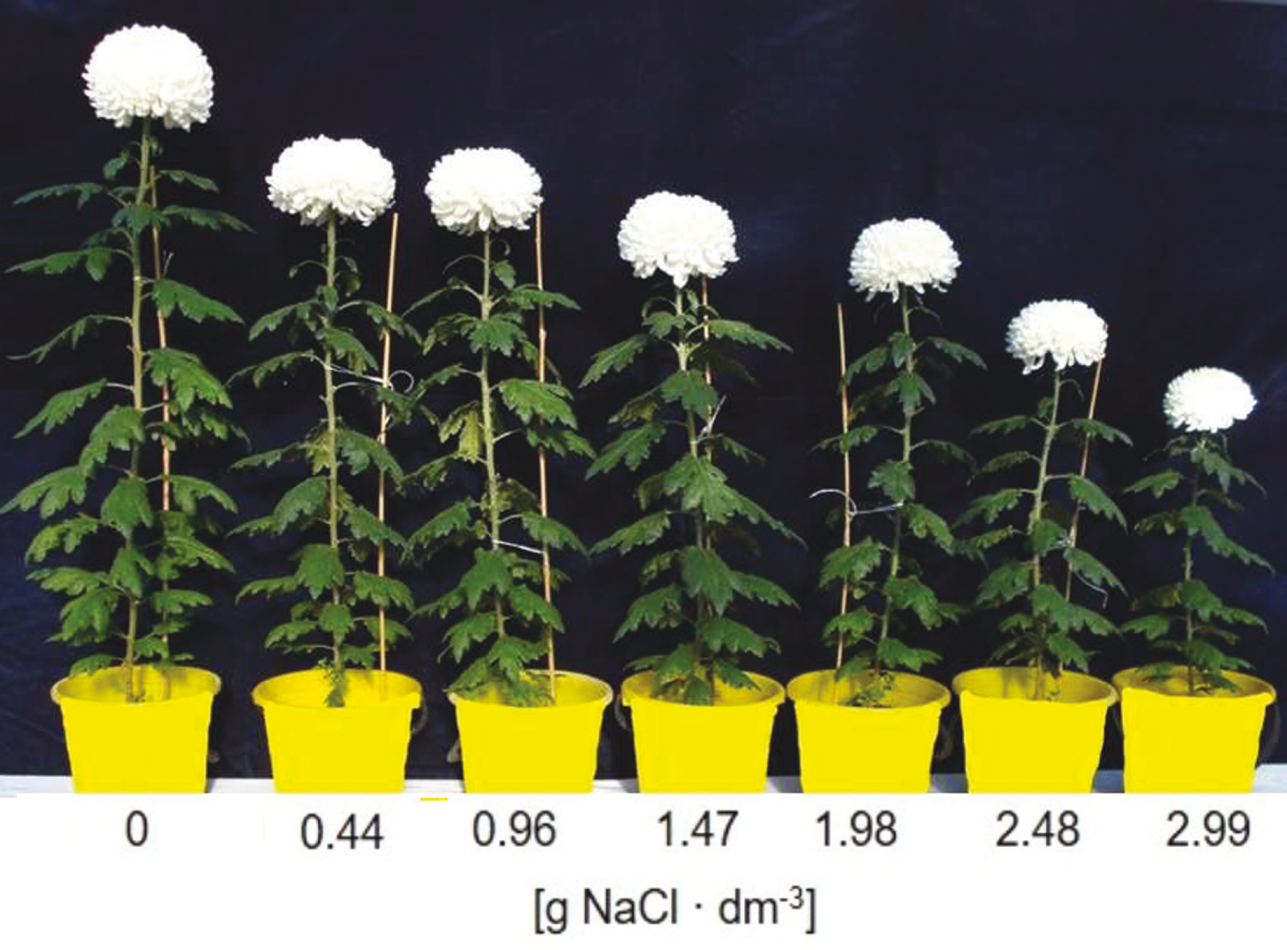
*Chrysanthemum × grandiflorum* ‘Palisade White’ at the end of experiment.

In both years a gradual and substantial reduction in all growth parameters was observed as NaCl dose increased (Figs 1 and 2). The average daily stem growth rate was reduced by about 4 % in both experiments for plants grown at the lowest salt dose (0.44 g dm^−3^), and by 42 % and 35 % for those grown at the highest dose (2.99 g dm^−3^) in experiment 1 and 2, respectively (Fig. 1D). As a result, a significant and gradual reduction in plant height was observed with the increase of NaCl concentration (Fig. 1C). Plant height was reduced by 3.8 % and by 6.4 % with the lowest salt dose in experiment 1 and 2, respectively. The highest NaCl dose caused an approximately 35 % decrease in plant height in both experiments. Plants cultivated with the lowest NaCl dose had in both years about 2–3 fewer leaves than the control (Fig. 1A). There were 15 and 12 fewer leaves in plants which grew on the substrate with the highest NaCl dose in experiment 1 and 2, respectively. Leaf area was reduced in both experiments by about 12 % and 50 % in plants grown with the lowest and highest NaCl dose, respectively (Fig. 1B).

**Figure 3.**
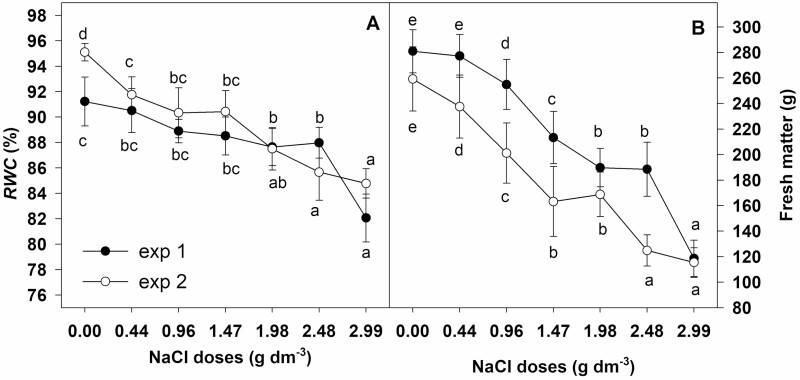
Effect of NaCl doses on (A) RWC and (B) fresh matter of aboveground parts of chrysanthemum. Values are expressed as a mean ±standard deviation. Different letters across salinity treatments indicate significant differences between means (fresh matter *n* = 20, RWC *n* = 5, Tukey test at *α* = 0.05).

Fresh matter and RWC were reduced gradually from the lowest to highest NaCl doses but in a slightly different way in the conducted experiments (Fig. 3). The changes in fresh matter (Fig. 3A) with the subsequent salt doses were generally consistent with the changes of leaf RWC (Fig. 3B). In experiment 2 significant reductions of fresh matter and RWC were observed in plants cultivated with the lower NaCl doses than in experiment 1. However, the highest NaCl dose (2.99 g dm^−3^) caused more than a 50 % reduction of fresh matter in both experiments and the decrease of RWC to 82.1 % in experiment 1 and to 84.8 % in experiment 2.

### Mineral concentration in leaves

**Figure 4.**
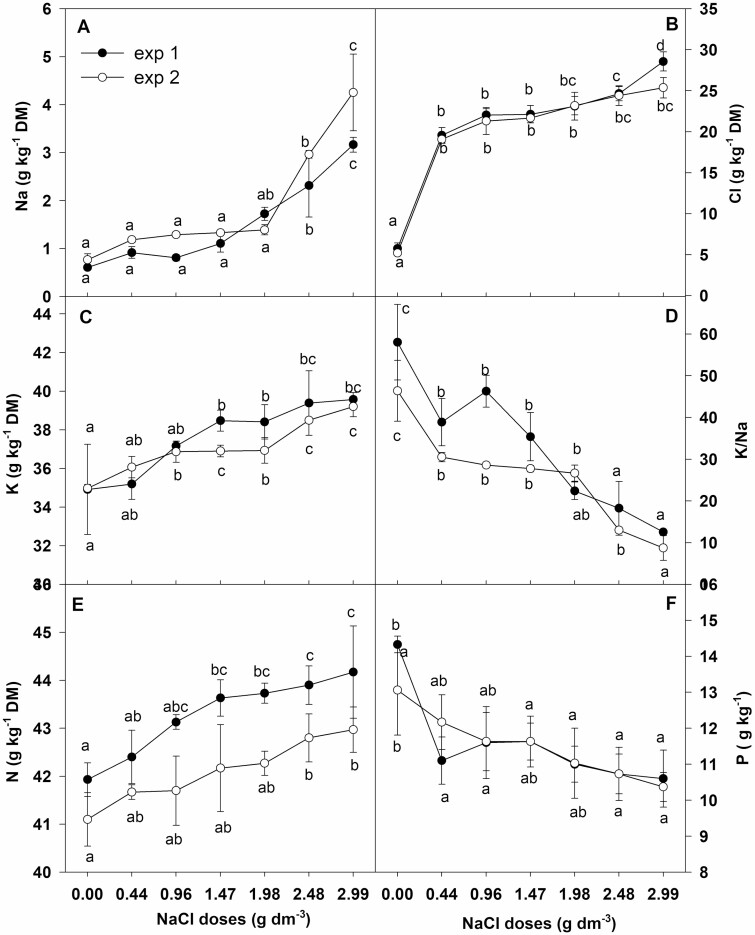
Effect of NaCl doses on (A) Na, (B) Cl, (C) K, (D) K/Na (E) N and (F) P concentrations (g·kg^−1^ DM) in leaves of chrysanthemum. Different letters across salinity treatments indicate significant differences between means (*n* = 3, Tukey test at *α* = 0.05).

In control plants Na concentration was in the range of 0.6–0.8 g kg^−1^ DM (Fig. 4A), which was much lower than Cl (5.2–5.8 g kg^−1^ DM) (Fig. 4B) and K (35 g kg^−1^ DM) concentration (Fig. 4C). The concentration of Cl^−^ increased by 3-fold at the lowest (0.44 g dm^−3^) and 5-fold at the highest NaCl dose (2.99 g dm^−3^) in both experiments. The Na^+^ level increased from 3- to 5-fold with NaCl doses of 1.98 g dm^−3^, 2.48 g dm^−3^ and 2.99 g dm^−3^ in experiment 1 and from 4- to 6-fold at NaCl doses of 2.48 g dm^−3^ and 2.99 g dm^−3^ in experiment 2. The level of K in both experiments increased gradually along with the increase of salt dose and was about 14 % higher at the highest NaCl dose as compared to the control. As a result of these changes, salt stress caused a reduction of K/Na ratio in chrysanthemum leaves from 58.2 to 12.4 in experiment 1 and from 43.8 to 8.0 in experiment 2 (Fig. 4D).

Salinity caused a dose-dependent significant increase of leaf N concentration from 41.93 to 44.17 g kg^−1^ DM in experiment 1 and from 41.1 to 42.9 g kg^−1^ DM in experiment 2 (Fig. 4E). However, foliar P concentration decreased across salinity and ranged from 10.60 to 14.13 g kg^−1^ DM in experiment 1 and from 10.4 to 13.1 g kg^−1^ DM in experiment 2 (Fig. 4F).

### Gas exchange parameters

**Figure 5.**
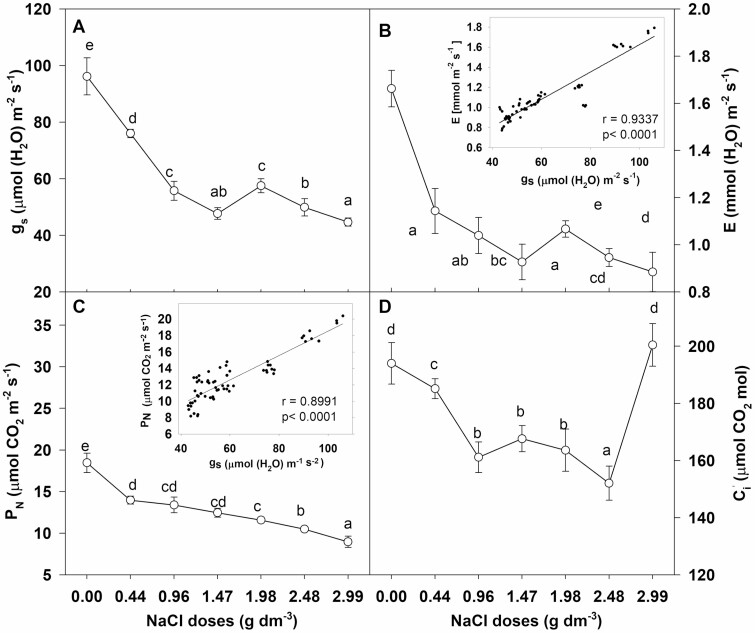
Effect of NaCl doses on (A) stomatal conductance (*g*_s_), (B) transpiration rate (E), (C) net photosynthetic rate (*P*_N_), and (D) intercellular CO_2_ concentration (*C*_i_) in leaves of chrysanthemum and linear regression between stomatal conductance and transpiration rate, stomatal conductance and net photosynthetic rate. Different letters across salinity treatments indicate significant differences between means (*n* = 9, Tukey test at *α* = 0.05).

Stomatal conductance and transpiration rate decreased when chrysanthemum plants were grown in the substrate with the addition of all NaCl doses (Fig. 5A and B). A statistically significant decrease of both parameters was observed in plants grown in substrate supplemented with the lowest NaCl dose (0.44 g dm^−3^) where stomatal conductance decreased by 21 % and the transpiration rate by 32 % compared to the control. Plants cultivated in substrate supplemented with at least 0.96 g dm^−3^ were characterized by about 43 % and 38 % reduction of stomatal conductance and transpiration rate, respectively. Salinity caused a dose-dependent decrease of the net photosynthetic rate (Fig. 5C). In the control plants the net photosynthetic rate was 18.45 μmol CO_2_ m^−2^ s^−1^ and was reduced by 25 % and 42 % in plants grown in substrate with the lowest (0.44 g NaCl dm^−3^) and highest (2.99 g NaCl dm^−3^) NaCl doses, respectively. Intercellular CO_2_ concentration was reduced by about 10 % and 17 % in plants grown at the lowest and medium NaCl doses (0.96–2.48 g dm^−3^), respectively (Fig. 5D), while in plants grown at the highest NaCl dose the intercellular CO_2_ concentration did not change. Both transpiration rate and net photosynthetic rate were dependent linearly on stomatal conductance (Fig. 5B and C).

### MI, chlorophyll and proline concentration

**Figure 6.**
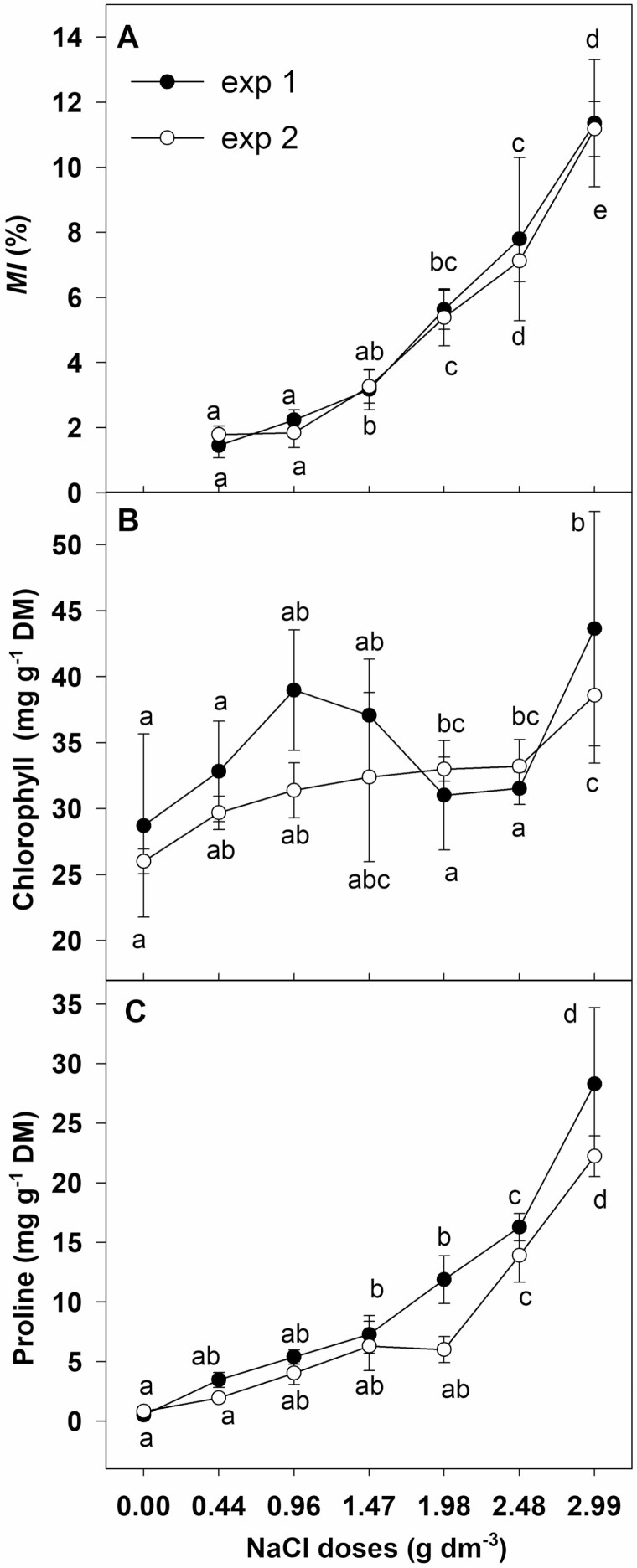
Effect of NaCl doses on (A) MI, (B) chlorophyll and (C) proline content in leaves of chrysanthemum. Different letters across salinity treatments indicate significant differences between means (*n* = 5, Tukey test at *α* = 0.05).

A gradual increase of MI with the increasing NaCl dose was found (Fig. 6A). In both experiments MI increased from about 2 % to about 11 % in plants cultivated with the lowest (0.44 g dm^−3^) and the highest (2.99 g dm^−3^) NaCl dose, respectively.

Leaf chlorophyll concentration in plants growing in substrate without NaCl addition was at the level of 28.7 µg g^−1^ DM and 26.0 µg g^−1^ DM in experiment 1 and 2, respectively (Fig. 6B). In both experiments the level of chlorophyll pigments concentration did not change in leaves of plants growing in substrate with the addition of salt in the range of 0.44–2.48 g NaCl dm^−3^. However, it increased by about 50 % and by 40 % in plants growing in substrate supplemented with the highest NaCl dose in experiment 1 and 2, respectively.

Proline concentration in control plants was at the level of 0.837 mg g^−1^ DM in experiment 1 and 0.507 mg g^−1^ DM in experiment 2, and it rose gradually with the increase of NaCl dose (Fig. 6C). Statistically significant increases of about 10- and 5-fold were observed in plants cultivated with a NaCl dose of 0.96 g dm^−3^ in experiment 1 and 2, respectively. Substrate supplementation with the highest NaCl doses caused a 55-fold increase of this amino acid in experiment 1 and a 27-fold increase in experiment 2.

## Discussion

Inhibition of growth has been observed in a large number of crop plants, such as *Ageratum*, *Tagetes* (Zapryanova and Atanassova 2009), *Pelargonium × hortorum* (Breś *et al.* 2016) and many other ornamental plants (Cassaniti *et al.* 2013; García-Caparrós and Lao 2018). Our results showed a significant reduction of growth parameters in ‘Palisade White’ chrysanthemum. Plants cultivated in saline substrate were smaller, characterized by a compact shape with fewer and smaller leaves than those grown without salt (Fig. 1). The higher the dose of added salt was, the greater was the observed reduction of growth parameters. According to Shannon and Grieve (1999), plant resistance to salt stress can be evaluated on the basis of two EC values. The salinity level causing the initial significant reduction of plant fresh matter is defined as the threshold EC value (EC_t_), whereas the EC_50_ value is the salinity level resulting in 50 % reduction of plant growth. Higher values of EC_t_ and EC_50_ indicate greater resistance to salt stress. In *Chrysanthemum × morifolium* ‘Yellow blush’ EC_t_ and EC_50_ of the substrate were 2 dS m^−1^ and 12.2 dS m^−1^, respectively (Lee and van Iersel 2008). What is more, the plant response to salinity depends on cultivation methods (soil, substrate, soilless culture) and salt application (regular or one-time application of salt solution). EC_t_ for *Chrysanthemum × morifolium* ‘Icecap’ cultivated in sandy soil and irrigated with seawater was 2.1 dS m^−1^. However, the EC level of 10.8 dS m^−1^, which was similar to EC_50_ of *Chrysanthemum × morifolium* ‘Yellow blush’, caused only a 8 % reduction of growth (Elhindi 2013). Our results showed that substrate EC_t_ for *Chrysanthemum × grandiflorum* ‘Palisade White’ was at the level of 1.4 and 0.9 dS m^−1^ in experiment 1 and 2, respectively. The reduction of fresh matter at the EC_t_ value was at the level of 9.3 % and 8.4 % in experiment 1 and 2, respectively. According to the Maas-Hoffman (1977) model of salt tolerance assessment we computed slope coefficient, by means of linear regression, which corresponds to the percent decrease of fresh matter per EC unit (dS m^−1^). This coefficient in both experiments was at the level of about 16 % per dS m^−1^, which indicates moderate sensitivity of chrysanthemum ‘Palisade White’ to salt stress.

The restriction of a plant’s ability to take up water under salt stress conditions triggers water deficit in leaves (Parihar *et al.* 2015). Our findings revealed a significant but slight decrease of water content in chrysanthemum and the highest NaCl dose caused only a mild water deficit in leaves. This mild water deficit led to a 50 % growth reduction although there was no decline in leaf N. Thus N deficiency could not be a reason for growth inhibition. The approximately 20 % decrease in P concentration revealed in our research could have a negative effect on biomass accumulation. A similar decrease in leaf P concentration and growth inhibition was noted in *C. morifolium* ‘Imperial Reagan’ and ‘Yellow blush’ (Warmenhoven and Baas 1995; Lee and van Iersel 2008). According to Lunt and Kofranek (1964) the critical P concentration for *C. morifolium* ranges from 2.6 to 11.5 g kg^−1^ DM. In leaves of *Dendranthema grandiflorum* (*C. morifolium*) ‘Jospithoven’ it varied according to the plant’s age and ranged from 0.7 g kg^−1^ DM at the initial vegetative stage to 19.3 g kg^−1^ DM at the flowering stage (Fernandes *et al.* 2012). Foliar P concentration in the examined *Chrysanthemum × grandiflorum* ‘Palisade White’ grown with the highest NaCl dose was at the level of about 10 g kg^−1^ DM.

Even a slight decrease in leaf RWC may reduce stomatal conductance (*g*_s_) and inhibit transpiration rate (*E*). The reduction in *g*_s_ restricts CO_2_ diffusion to leaf tissues and affects the inhibition of photosynthetic rate (*P*_N_) as well as growth cessation (Stepien and Johnson 2009; Shongwe and Wahome 2014). Significant growth inhibition without changes in leaf water content as well as a decrease in *g*_s_ and *P*_N_ was observed in salt-stressed calla lilies (Veatch-Blohm *et al.* 2012). The results of our research revealed significant and dose-dependent inhibition of *P*_N_ measured on the basis of the amount of assimilated CO_2_, along with a decrease in *g*_s_, which led to the restriction of CO_2_ diffusion to leaves. A decrease of intercellular CO_2_ concentration (*C*_i_), more than 20 % inhibition of *P*_N_ and growth restriction were observed in chrysanthemum cultivated in substrate supplemented with NaCl doses from 0.44 to 2.48 g dm^−3^. However, in plants cultivated with the highest NaCl dose (2.99 g dm^−3^), *C*_i_ remained at the same level as in controls, while *g*_s_ and *P*_N_ were significantly reduced. This indicates that the inhibition of photosynthesis in these plants was not caused by the restriction of CO_2_ availability. Similarly, reduction of *g*_s_ and inhibition of *P*_N_ were observed in *Shepherdia argentea* under severe salt stress conditions (600 mM NaCl), despite high *C*_i_, and in *D. grandiflorum* at a lower (200 mM NaCl) salinity level (Qin *et al.* 2010; Wang *et al.* 2017). Moreover, the increase of internal CO_2_ concentration caused only partial recovery of *P*_N_ in salt-stressed plants (Van Zelm *et al.* 2020). These findings show that inhibition of *P*_N_ caused by salt stress could have been affected both by a reduced amount of CO_2_ available for fixation and by a stomatal closure-independent effect. Stomatal limitation of photosynthesis mainly occurs in short-term salinity stress (Garcia-Caparrós and Lao 2018). Long-term salt stress causes the accumulation of large amounts of toxic Na and Cl in cells, which adversely affects cell metabolism and growth (Munns and Teste 2008; Liang *et al.* 2018). Leaves of salt-stressed *Chrysanthemum × morifolium* ‘Imperial Reagan’ showed a several-fold increase of these ions, but the level of sodium increased more than chlorine (Warmenhoven and Baas 1995). The results presented in this paper revealed similar (6- to 7-fold) increases of both Na and Cl concentrations in leaves of chrysanthemum cultivated with the highest NaCl dose. Indeed, the level of Na increased several fold with the highest NaCl dose but the concentration in leaves reached a level not greater than 5 g kg^−1^ DM. However, the critical harmful leaf Na concentration in *C. morifolium* was at the level of 8.8 g kg^−1^ DM in ‘Flirt’, 6.45 g kg^−1^ DM in ‘Jayanti’ and 18.7 g kg^−1^ DM in ‘Yellow blush’ (Lee and van Iersel 2008; Rahi and Singh 2011). The harmful concentration of Cl for sensitive species is in the range of 4–7 g kg^−1^ DM and 15–50 g kg^−1^ DM for resistant species (Teakle and Tyerman 2010). In leaves of *Chrysanthemum × grandiflorum* ‘Palisade White’ cultivated at the highest NaCl dose the level of Cl increased to about 30 g kg^−1^ DM without causing any leaf burning or plant death. High concentrations of Cl can have a harmful effect on photosynthesis through their impact on the integrity of cell membranes and chlorophyll degradation; they bring about destruction of chloroplast pigment protein complexes and damage of the Photosystem II antenna system as well as inhibition of ribulose bisphosphate carboxylase activity (Teakle and Tyerman 2010; Tavakkoli *et al.* 2011; Parihar *et al.* 2015). However, the lack of changes in leaf chlorophyll content in the examined chrysanthemum plants at salinity levels from 0.44 to 2.48 g NaCl dm^−3^ and the increased level at the highest NaCl dose (2.99 g dm^−3^) suggest that the reduction of the photosynthetic rate was not caused by a decrease in the content of these pigments. Salt stress caused dose-dependent membrane damage in chrysanthemum plants. The slight decrease in RWC, even in plants grown in substrate supplemented with the highest NaCl dose, and about 11 % MI, indicates a direct harmful effect of high concentrations of Cl^−^, rather than tissue dehydration, on membrane integrity, which may affect the photosynthetic machinery. Hence, the restriction of CO_2_ assimilation evidenced by the inhibition of photosynthesis and the maintenance of high *C*_i_ despite more than a 60 % decrease of stomatal conductance could be caused by the harmful effect of Cl^−^ on the photosynthetic machinery (non-stomatal effect), leading to the decline of *P*_N_ and growth restriction.

High external Na^+^ concentration under salt stress conditions often affects competitive inhibition of K^+^ uptake, leading to its deficiency (Tavakkoli *et al.* 2011; Assaha *et al.* 2017). In the examined *Chrysanthemum × grandiflorum* ‘Palisade White’, the level of K increased and was about 14 % higher at the highest NaCl dose compared to the control. This indicates the undisturbed K^+^ uptake even at the highest NaCl dose. The capacity to prevent restriction of K^+^ uptake and K^+^ retention ability was shown in salt-tolerant accessions of *Arabidopsis* (Sun *et al.* 2015). However, better salt tolerance of *Chrysanthemum chinense* than *C. morifolium* was likely caused by the ability to avoid accumulation of Na and reduction of the K level in plant tissue, probably due to the existence of a selective ion transport system (Gao *et al.* 2016). In salt-stressed spinach preferential K^+^ absorption over Na^+^ was observed due to membrane-bound transport proteins (Ferreira *et al.* 2020). Foliar K concentration in salt-stressed *Chrysanthemum × grandiflorum* ‘Palisade White’ was about 10-fold higher than Na concentration, which may show on the limitation of Na^+^ uptake. It was shown that downregulation of membrane proteins involve in avoidance of excessive Na^+^ absorption (cyclic nucleotide-gated channels) in roots of a salt-tolerant rice cultivar prevented the influx of Na^+^ (Assaha *et al.* 2017). In relatively salt-tolerant ornamental shrubs, i.e. *Cestrum fasciculatum*, *Escallonia rubra* and *Viburnum lucidum*, less Na than Cl was accumulated in leaves which probably indicates a limited transport of Na^+^ to these organs (Cassaniti *et al.* 2009). High Cl level can competitively inhibit the uptake of NO_3_^−^ in salt-stressed plants and cause nitrogen starvation or deficiency (Guo *et al.* 2017). However, significant accumulation of Cl in the leaves of chrysanthemum ‘Palisade White’ did not affect the decrease of N concentration, which indirectly indicates on the maintenance of NO_3_^−^ uptake.

Proline accumulation is a well-known resistance response to salt stress in crop plants (Per *et al.* 2017; Moukhtari *et al.* 2020). However, there are limited literature data focused on the effects of salt stress on the accumulation of proline in ornamental plants, with regard to the role of this amino acid in salinity resistance. Zheng *et al.* (2015) found that exogenous application of proline resulted in the improvement of salinity resistance in *Eurya emarginata.* Salt resistance of *C. morifolium* germplasm was due to enhanced proline and chlorophyll levels as well as better shoot and root growth (Vanlalruati *et al.* 2019). About a 30 % increase of proline level was observed in leaves of salt-stressed pelargonium, along with only a slight reduction in the maximum quantum yield of PSII and no reduction in the efficiency of PSII (Breś *et al.* 2016). The present data show a dose-dependent increase of proline in leaves of ‘Palisade White’ chrysanthemum. The level of this amino acid increased about 5- to 10-fold in plants cultivated with the lower NaCl doses but 27- to 55-fold in those which grew on the substrate with the highest dose of salt. Such high proline accumulation could have an effect on the alleviation of leaf dehydration as well as on chlorophyll depletion, which resulted in a slight decrease of leaf RWC and no reduction of chlorophyll level. A high proline level in salt-stressed chrysanthemum could also help the plant to avoid a potentially toxic impact of Cl, which was manifested as no leaf burning or plant death. Such a beneficial effect has been evidenced by some research in plants treated with exogenous proline (Moukhtari *et al.* 2020 and references therein). What is more, bearing in mind the positive effect of exogenous proline on the uptake of N and K under salinity (Alam *et al.* 2016), it can be supposed that the undisturbed uptake of these elements in *Chrysanthemum × grandiflorum* ‘Palisade White’ could have been the result of substantial accumulation of this amino acid.

In conclusion, our findings indicate that based on the Maas and Hoffman (1977) model, the ‘Palisade White’ chrysanthemum may be considered as moderately salt sensitive in terms of growth inhibition. However, the long-term salt stress affected dose-dependent growth restriction but caused only a minor decrease of leaf hydration and no signs of damage, such as chlorophyll depletion, leaf browning or necrotic spots, were observed for any of the used salt concentrations. The adverse effect of salt stress was probably counteracted by high proline accumulation which improved RWC, alleviated the toxic effect of chloride and maintained the undisturbed uptake of potassium and other minerals. The obtained results also revealed that limitation of photosynthesis was probably the main reason that salinity caused growth inhibition.

## Supporting Information

The following additional information is available in the online version of this article—

Table S1. Data set of growth parameters.

Tables S2 and S2a. Data set of physiological and biochemical traits.

Table S3. Data set of gas exchange parameters.

plac015_suppl_Supplementary_Table_S1

plac015_suppl_Supplementary_Table_S2

plac015_suppl_Supplementary_Table_S2a

plac015_suppl_Supplementary_Table_S3

## Data Availability

All data are available as Supporting Information in the online version of this article.

## Notes

**Phenome, Genome & Environment. Chief Editor**: Colleen Doherty
